# FK506 binding protein 51 positively regulates melanoma stemness and metastatic potential

**DOI:** 10.1038/cddis.2013.109

**Published:** 2013-04-04

**Authors:** S Romano, S Staibano, A Greco, A Brunetti, G Nappo, G Ilardi, R Martinelli, A Sorrentino, A Di Pace, M Mascolo, R Bisogni, M Scalvenzi, B Alfano, M F Romano

**Affiliations:** 1 Department of Molecular Medicine and Medical Biotechnologies, Federico II University, Naples, Italy; 2 Dipartimento di Scienze Biomediche Avanzate, Federico II University, Naples, Italy; 3 CEINGE Biotecnologie Avanzate, Naples, Italy; 4 Department of Medicine and Surgery, University of Salerno, Salerno, Italy; 5 Department of Dermatology, University Federico II of Naples, Naples, Italy; 6 Institute of Biostructure and Bio-Imaging, National Research Council (CNR), Naples, Italy

**Keywords:** melanoma, metastasis, cancer stem cell, epithelial to mesenchymal transition, FKBP51

## Abstract

Melanoma is the most aggressive skin cancer; there is no cure in advanced stages. Identifying molecular participants in melanoma progression may provide useful diagnostic and therapeutic tools. FK506 binding protein 51 (FKBP51), an immunophilin with a relevant role in developmental stages, is highly expressed in melanoma and correlates with aggressiveness and therapy resistance. We hypothesized a role for FKBP51 in melanoma invasive behaviour. FKBP51 promoted activation of epithelial-to-mesenchymal transition (EMT) genes and improved melanoma cell migration and invasion. In addition, FKBP51 induced some melanoma stem cell (MCSC) genes. Purified MCSCs expressed high EMT genes levels, suggesting that genetic programs of EMT and MCSCs overlap. Immunohistochemistry of samples from patients showed intense FKBP51 nuclear signal and cytoplasmic positivity for the stem cell marker nestin in extravasating melanoma cells and metastatic brains. In addition, FKBP51 targeting by small interfering RNA (siRNA) prevented the massive metastatic substitution of liver and lung in a mouse model of experimental metastasis. The present study provides evidence that the genetic programs of cancer stemness and invasiveness overlap in melanoma, and that FKBP51 plays a pivotal role in sustaining such a program.

Melanoma is a dynamic and heterogeneous skin neoplasia with adverse prognosis in advanced stages. Despite the large body of studies published during the past decade that have contributed to the advance of knowledge in this field,^1, 2, 3, 4^ the complex biology of this tumour remains obscure. Identifying molecular players with a determinant role in tumour progression may provide useful diagnostic and therapeutic tools and greatly improve treatment outcomes, thereby changing the fate of this unpredictable tumour. We have recently shown that FK506 binding protein 51 (FKBP51), a protein involved in cancer chemoresistance^5, 6, 7, 8, 9, 10, 11^ is a promising target for radiosensitization strategies in melanoma.^8^ FKBP51 is highly expressed in melanoma.^5, 8^ Such expression correlates with tumour aggressiveness and is maximal in metastatic lesions.^8^ Moreover, FKBP51 overexpression was apparently associated with a melanoma cancer stem cell (MCSC) phenotype.^8^FKBP51 (FKBP5 official symbol) is a large molecular weight component of the family of FK506 binding proteins (FKBPs), classically known as the intracellular receptors for immunosuppressants FK506 and rapamycin.^12, 13^ FKBPs are multifunctional proteins that modulate several signal transduction pathways.^12, 13, 14, 15^ Although the knowledge of FKBP structure and function is far from complete, recent reports reveal that this class of proteins affects gene expression, DNA repair, and DNA replication (reviewed in Yao *et al.*^15^). These functions are mediated by histone chaperone activity, regulation of transcription factors, and modifications of chromatin structure.^15^ In accordance with these concepts, FKBP51 plays a relevant role in developmental stages in both mammals^16, 17, 18^ and other organisms.^19^ FKBP51 is among the top candidate genes expressed in mesenchymal stem cells,^18^ particularly during the mitotically active phase that precedes differentiation into the three mesodermal lineages, namely osteogenesis/chondrogenesis/adipogenesis.^16, 17^ Similarly, *PAS-1* (PASTICCINO1 gene), the FKBP51 homologue in Arabidopsis, plays a critical role in the growth and differentiation of this organism.^19^Recent breakthroughs suggest that genes active in developmental processes are relevant to cancer biology and play a determinant role in reprogramming cancer cells to activate stemness.^20^ Notably, cancer stem cells (CSCs) have the ability to self-renew, as well as to differentiate into more mature cancer cells. This conversion is bidirectional within certain limits, resulting in dynamic variation of CSC abundance and heterogeneity of the tumour due to the formation of diverse subpopulations that support tumour growth in different ways.^20^ Epithelial-to-mesenchymal transition (EMT) is a normal process in embryonic development in which epithelial cells transform into mesenchymal cells, the highly mobile cells that give rise to bone, muscle, connective tissue, and blood vessels.^21^ Cancer cells require EMT to spread and metastasize.^22^ Induction of the EMT transdifferentiation programme in cancer cells enables them to not only disseminate from primary tumours and form metastases, but also to acquire self-renewal capability (i.e., CSC phenotype), which is crucial to their subsequent expansion at sites of dissemination.^22, 23^ We hypothesized a role for FKBP51 in the regulation of the melanoma stemness phenotype and invasive behaviour.

## Results

### FKBP51 positively regulates EMT hallmarks in melanoma

**Figure 1:**
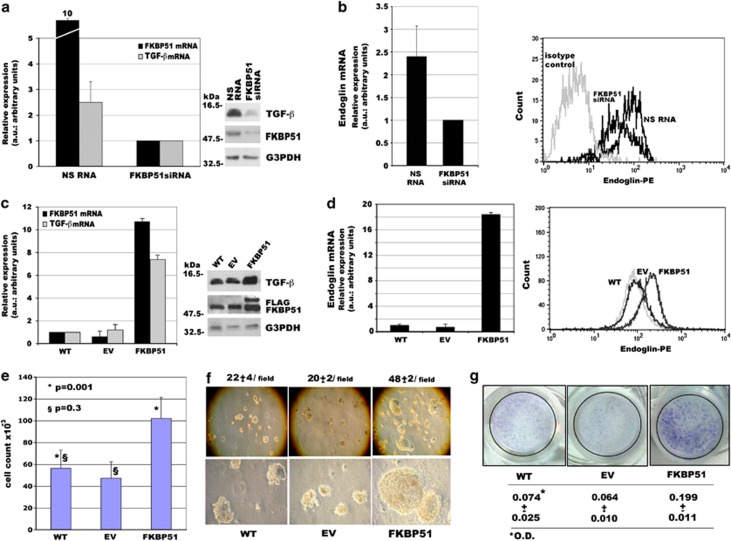
FKBP51 modulates EMT hallmarks. (**a** and **b**) FKBP51 silencing of SAN melanoma cells decreases TGF-*β* and T*β*RIII expression. Normalized expression rates (mean±S.D.) of TGF-*β* (**a**, left) and T*β*RIII (**b**, left) mRNA levels; (**a**, *n*=3; **b**, *n*=5). FKBP51 siRNA-treated sample expression=1. Western blot assay of TGF-*β* in SAN melanoma cells treated with nonsilencing (NS) RNA or FKBP51 siRNA (**a**, right). Flow cytometric analysis of T*β*RIII (endoglin) expression (**b**, right): FKBP51 siRNA-treated cells moved to the left relative to NS RNA-treated cells because of reduced staining (MFI: 111±10 and 64±3 in NS RNA and FKBP51 siRNA-treated cells, respectively). **(c**, and **d**) FKBP51 overexpression enhances TGF-*β* and T*β*RIII expression. Normalized expression of TGF-*β* (**c**, left) and T*β*RIII (**d**, left) mRNA in WT, EV-, or FKBP51-stably transfected melanoma cells. WT sample expression=1. (**c**, *n*=3; **d**, *n*=5). Western blot assay of TGF-*β* levels in the same cells (**c**, right). Anti-Flag-labelled exogenous FKBP51. Flow cytometric analysis of T*β*RIII expression (**d**, right). FKBP51-overexpressing cells moved to the right relative to WT or EV cells, indicating increased expression. (**e**) Graphic representation of cell counts ( × 10−3) in WT, EV-, or FKBP51-stably transfected melanoma cell cultures. Results are means of six independent experiments each performed in triplicate. (**f**) FKBP51 upregulates colony number and size. Colonies were counted under low magnification ( × 10) at four points on each dish. The data represent the means of two independent experiments performed in triplicate. Magnification: upper, × 10; lower × 40. (**g**) Boyden chamber filters invaded by melanoma cells. Filter was stained with Crystal Violet. OD value was obtained by reading the eluted Crystal Violet with a spectrophotometer at a wavelength of 570 nm. The increased OD in FKBP51 samples indicated that FKBP51-overexpressing cells invaded matrigel more efficiently than control cells. The data represent the means of three independent experiments performed in triplicate

The transforming growth factor-*β* (TGF-*β*) is a key regulator of EMT.^24, 25, 26^ Furthermore, a critical role in melanoma tumour cell plasticity is covered by the type III TGF-*β* receptor (T*β*RIII or betaglycan or endoglin).^27^ We measured the mRNA and protein levels of TGF-*β* and T*β*RIII in FKBP51-depleted or FKBP51-overexpressing SAN melanoma cells. TGF-*β* protein was investigated with western blot (Figures 1a and c; right) and T*β*RIII protein was investigated with flow cytometry (Figures 1b and d; right). FKBP51 silencing resulted in reduced TGF-*β* expression (Figure 1a). Conversely, enhancement of TGF-*β* expression was observed in FKBP51-overexpressing SAN melanoma cells (Figure 1c). T*β*RIII levels were modulated in a similar manner (Figures 1b and d). FKBP51-overexpressing cells showed an increased proliferation (Figure 1e). We performed soft agar colony formation assays to investigate the capability of FKBP51-overexpressing melanoma cells to promote anchorage-independent growth, a typical feature of EMT.^8^ Figure 1f shows that FKBP51 strikingly stimulated soft agar growth, as suggested by the colony number and size that were remarkably increased in FKBP51-overexpressing cells. In addition, using Boyden chamber and matrigel,^28^ we found that the ability of melanoma cells to migrate through transwell filters and invade matrigel was enhanced by FKBP51-overexpression (Figure 1g). As expected, invasion was remarkably reduced in FKBP51-silenced melanoma (Supplementary Figure S1). Overall, our findings suggested that FKBP51 promotes activation of EMT features in melanoma. We confirmed that FKBP51 knockdown resulted in TGF-*β* and T*β*RIII decrease in A375 melanoma cell line also, using a different small interfering RNA (siRNA) (Supplementary Figure S2).

### FKBP51 regulates expression of melanoma stemness markers

**Figure 2:**
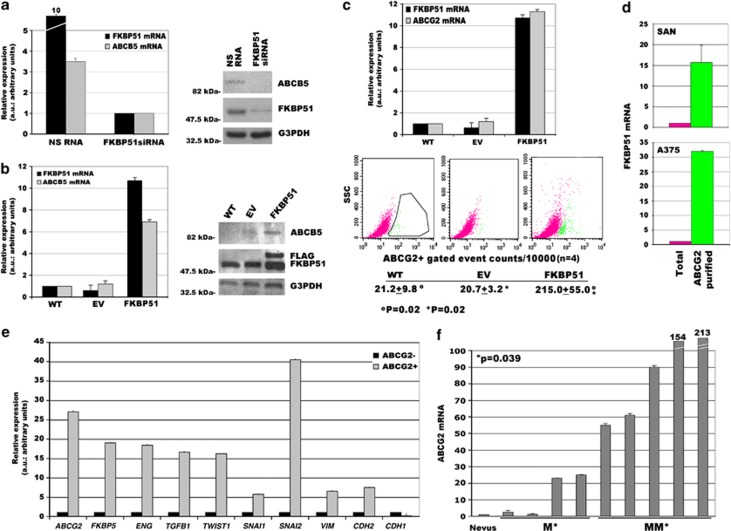
FKBP51 increases expression of melanoma CSC markers. (**a**) FKBP51 silencing decreases ABCB5 levels. (Left) Normalized expression rates (arbitrary units (AU)) (mean±S.D.) of FKBP51 (black) and ABCB5 mRNA (grey) (*n*=3). FKBP51-treated sample expression=1. (Right) Western blot assay of ABCB5 and FKBP51 levels. (**b**) FKBP51 overexpression enhances ABCB5 levels. (Left) Normalized expression of FKBP51 mRNA (black) and ABCB5 mRNA (grey) measured in WT, EV-, or FKBP51-stably transfected melanoma cells. WT sample expression=1; *n*=3. (Left) Western blot assay of ABCB5 and FKBP51 levels in the same cells. Anti-Flag-labelled exogenous FKBP51. (**c**) ABCG2+ melanoma cells increase in FKBP51-overexpressing cells. (Upper) Normalized expression of FKBP51 mRNA (black) and ABCG2 mRNA (grey) measured in WT, EV-, or FKBP51-stably transfected melanoma cells. WT sample expression=1; *n*=5. (Lower) Flow cytometric histograms of ABCG2 expression (green population); mean±S.D. of counts are shown. (**d**) Enhanced FKBP51 mRNA levels in sorted ABCG2+ melanoma cells (SAN, upper; A375, lower). Whole cell expression=1; (*n*=3). (**e**) Enhanced EMT gene transcripts in sorted ABCG2+ melanoma cells. ABCG2− sample expression=1; *n*=3. (**f**) Expression of ABCG2 transcript in 9 deparaffinized tumours, 4 primary melanoma (M) samples, and 5 metastases (MM). A naevus sample was arbitrarily chosen with expression=1

Normal stem cells have high drug efflux capability because of the expression levels of ATP binding cassette (ABC) transporters, which actively pump drugs out of the cell. ABC, subfamily B, member 5 (ABCB5)^29^ and ABC, subfamily G, member 2 (ABCG2)^30^ are members of this superfamily of proteins, and are known molecular determinants of cancer stem/initiating cells of melanoma.^29, 30^ We investigated if FKBP51 modulated MCSC phenotype. To this end, we silenced FKBP51 with siRNA and measured mRNA levels of ABCB5 using real-time PCR. FKBP51 siRNA produced a 10-fold decrease in FKBP51 mRNA levels (black histogram) and a threefold decrease in ABCB5 mRNA (grey histogram), compared with cells transfected with a nonsilencing (NS) RNA (Figure 2a, left). We confirmed that FKBP51 knockdown resulted in ABCB5 decrease in several melanoma cell lines, using a different siRNA (Supplementary Figure S3). Conversely, in SAN melanoma cells overexpressing FKBP51, a sevenfold increase in ABCB5 mRNA (grey histogram) was measured (Figure 2b; left), compared with wild-type (WT) or empty-vector stably transfected cell (EV) mRNA levels. Similarly, ABCB5 expression levels, measured by western blot, decreased and increased in accordance with FKBP51 levels (Figures 2a and b; right). In the same way, ABCG2 mRNA was upregulated in melanoma cells that over expressed FKBP51 (Figure 2c, upper). Most interestingly, flow cytometry demonstrated that the cell count of ABCG2-positive cells was increased 10-fold when FKBP51 was overexpressed (Figure 2c, lower). Furthermore, ABCG2+ sorted cells from two different cell lines, SAN and A375 (Figure 2d, upper and lower, green histogram), expressed very high levels of FKBP51 mRNA compared with unsorted cells (Figure 2c, pink histogram). These results suggested that FKBP51 upregulates the melanoma stemness phenotype. Additional clones were analysed for TGF-*β*, T*β*RIII, and stemness marker expression (Supplementary Figure S4). To confirm the stemness feature of ABCG2+ sorted cells, we performed a genome-wide study of cancer stem cell gene expression on these cells using a stem cell real-time PCR array (RT2 profiler PCR Array-Human Stem Cell signalling pathway; Qiagen Sciences, Philadelphia, PA, USA). ABCG2+ cells expressed increased levels (more than twofold increase) of 23 stem cell gene transcripts, including Notch, Wnt, and bone morphogenetic protein (Supplementary Figure S5). As FKBP51 has shown to positively regulate EMT, and ABCG2+ melanoma cells contained high FKBP51 levels, we hypothesized that ABCG2+ cells expressed EMT genes. To verify this hypothesis, we measured, the expression of TGF-*β*, endoglin, and other EMT genes (*VIM, TWIST1, SNAI1, SNAI2, CDH2*)^31, 32, 33, 34^ in sorted ABCG2+ melanoma cells. These cells exhibited expression levels of EMT genes enhanced by 5- to 40-fold compared with ABCG2− cells (Figure 2e). As expected, *CDH1* expression was reduced. These findings suggested that MCSC and EMT genetic programs were simultaneous active in ABCG2+ cells. The strict relationship between MCSCs and metastasis was further reinforced by the finding that deparaffinized melanoma metastasis samples contained ABCG2 levels significantly higher than primary melanoma samples. A naevus sample was arbitrarily chosen as reference sample (expression=1) (Figure 2f). This finding is in accordance with the concept that metastasis requires activation of MCSC genes to spread and generate secondary tumours.^20, 22, 23^

### Invading melanoma cell coexpresses stemness markers and FKBP51

**Figure 3:**
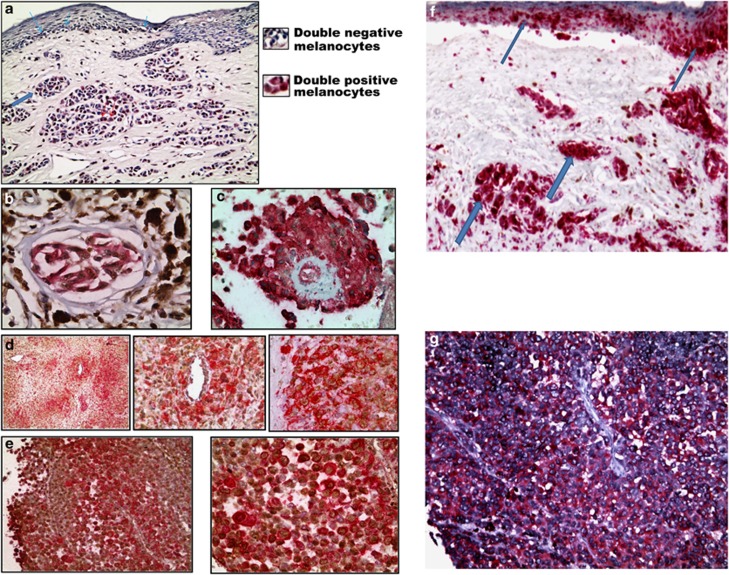
Malignant invasive melanocytes are double-stained with nuclear FKBP51(brown) and cytoplasmic/membranous nestin (red). (**a**) FKBP51/nestin staining of a primary melanoma: blue arrows: malignant melanocytes, negative for FKBP51 and nestin (thin arrows: radial, noninvasive growth phase; thick arrows: vertical, invasive growth phase); red arrows: malignant melanocytes double immunostained with FKBP51 and nestin, in vertical, invasive growth phase of melanoma (IHC, × 100). Malignant melanocytes negative for FKBP51 and nestin are detectable in both radial and vertical growth phases. FKBP51/nestin double-stained malignant melanocytes are observed in the vertical growth phase. Enlarged details of double-negative and double-positive melanocytes are shown on the right. (**b**) Neoplastic embolus in a dermal vessel of skin melanoma containing double-stained melanocytes (IHC: × 400); melanin pigment is visible outside of the vessel. (**c**) Vascular invasion of double-stained melanoma cells in brain metastasis (IHC, × 400). (**d**) Melanoma brain metastasis, FKBP51/nestin-positive cells form aggregates with a preferentially perivascular localization (IHC: × 50, × 100, × 400). (**e**) Brain metastasis, the cerebral tissue is completely substituted by double-stained malignant melanocytes (IHC: × 100, × 200). (**f**) The same case showed in (**a**), malignant melanocytes are immunostained with MART-1. Radial growth phase: thin blue arrows; vertical growth phase: thick blue arrows. (**g**) A serial section of the case showed in (**e**), malignant melanocytes are immunostained with MART-1

To address the relationship between FKBP51, stemness, and metastasis in melanoma patients, we performed double immunochemical staining of FKBP51 and nestin, an intermediate filament expressed in the cytoplasm of neuroepithelial stem cells^35^ in a series of 10 primary cutaneous and 20 brain melanoma metastases. We found melanoma cells with nuclear FKBP51 and membrane–cytoplasmic nestin in the vertical (invasive, intradermal) growth phase of primary melanomas. Figure 3a shows a representative primary melanoma. The observation that neoplastic emboli of dermal vessels in primary tumours (Figure 3b) and vascular invasions (Figure 3c) were mainly constituted by such cells suggested that these double-stained melanoma cells had capability of extravasation and surviving within the circulation. In addition, double-stained melanoma cells were found abundant in metastatic solid aggregates in the brains of patients with advanced melanoma, whether perivascular (Figure 3d) or completely substituting the tissue (Figure 3e). In (Figures 3f and g), serial sections of the same cases shown in (Figures 3a and e), respectively, were immunostained with MART-1 (melanoma antigen recognized by T cells). These findings support the conclusion that double-positive melanoma cell is endowed with metastatic capacity.

### FKBP51 targeting prevents organ colonization in mouse model of melanoma metastasis

**Figure 4:**
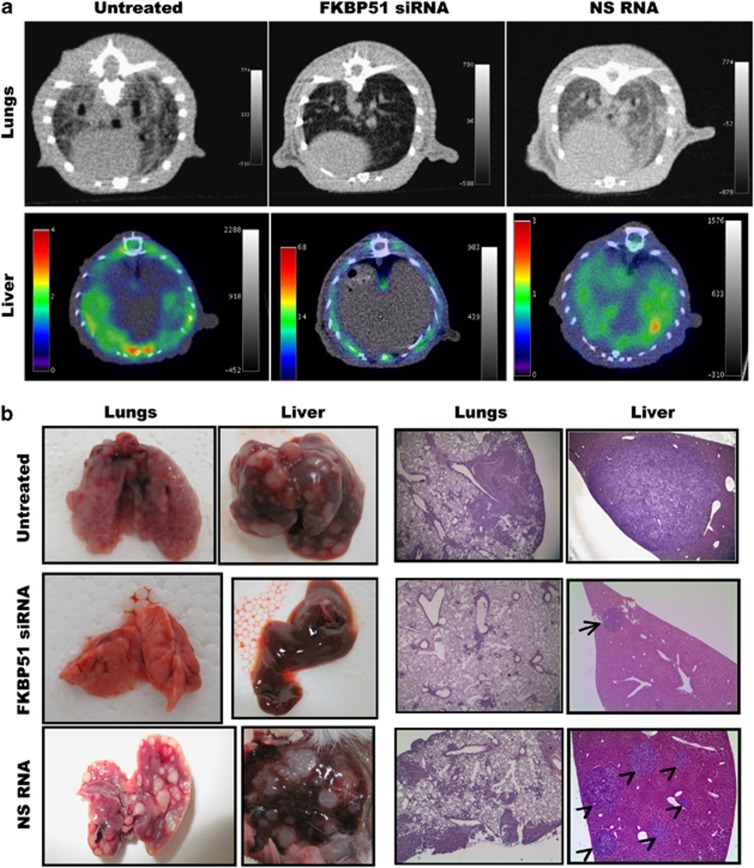
FKBP51 siRNA counteracts metastasis formation. (**a** upper) CT images of lungs axial view. In the untreated and NS RNA-treated mouse, marked thickening of the lung interstitium and peribronchial tissue; in the FKBP51 siRNA-treated mouse, lung parenchyma shows no significant abnormality. (**a** lower) ([18]F-FDG PET/CT images of liver, axial view. Increased FDG uptake throughout untreated or NS RNA-treated liver. No FDG uptake in right or left FKBP51 siRNA-treated liver lobes. (**b**, left) Representative lungs and liver excised from mice. Organs from untreated or NS RNA-treated mice were completely substituted by nodules. Organs from FKBP51 siRNA-treated mice were apparently not altered morphologically. (**b** right) Haematoxylin and eosin (H/E) staining of lung and liver biopsies. Lung specimens (H/E; × 50): large metastatic melanoma nodules extensively involve the lung specimen from untreated mouse and NS RNA mouse; in lung biopsy from a FKBP51 siRNA-treated mice, metastatic cells form small peribronchial and perivascular aggregates. Liver specimens (H/E; × 25): the metastasis extensively substitutes the liver tissue in untreated mouse; only a single small subcapsular nodule is visible in liver biopsy from FKBP51 siRNA-treated mouse; NS RNA mouse liver is completely substituted by multiple nodules

To confirm the role of FKBP51 in melanoma invasion and progression, we utilized an experimental metastasis model of melanoma that resembles the last steps of tumour cell spontaneous metastasis, including survival of malignant cells in the circulation, extravasation, and colonization in the distal organs. Injecting SAN melanoma cells into the tail vein of IL2-NOD-SCID mice produced prominent multifocal lesions in lungs and liver after 28 days. A previous *in vivo* study has shown the specificity and efficacy of FKBP51 siRNA in reducing levels of FKBP51 persistently in tumour xenografts.^8, 36^ The siRNA efficacy and specificity is shown in Supplementary Figure S6. We investigated the effect of FKBP51 targeting on melanoma colonization of mouse organs. Two separate doses of FKBP51 siRNA, or NS RNA as control, were systemically administered to the mice 15 and 21 days after the injection of SAN cells. Organ colonization was evaluated in both live animals and post mortem. FKBP51 siRNA dramatically reduced metastasis formation. Computerized tomography (CT) imaging of lungs showed marked thickening of the lung interstitium and peribronchial tissue in the untreated mice or mice treated with NS RNA; in the FKBP51 siRNA-treated mouse, lung parenchyma showed no significant abnormality (Figure 4a, upper). Qualitative positron emission tomography (PET)/CT image analysis demonstrated high Fluorodeoxyglucose (18F) ([18]F-FDG) uptake in the liver of untreated mice or mice treated with NS RNA. In contrast, low [18]F-FDG uptake limited to small areas was observed in the liver of FKBP51 siRNA-treated mouse (Figure 4a, lower). Quantitative analysis performed on [18]F-FDG PET/CT liver images revealed that mean standardized uptake values (SUVs) of FKBP51 siRNA-treated mice (0.16±0.04 for right liver and 0.19±0.07 for left liver) were significantly lower (*P*=0.003 and *P*=0.03 for right and left liver, respectively) than SUVs of mice treated with control RNA (0.55±0.05 and 0.70±0.10), and significantly lower (*P*=0.002 and *P*=0.001) than SUVs from untreated mice (0.65±0.10 and 0.80±0.20). There was no difference between SUVs of mice receiving NS RNA and SUVs of untreated mice.

**Figure 5:**
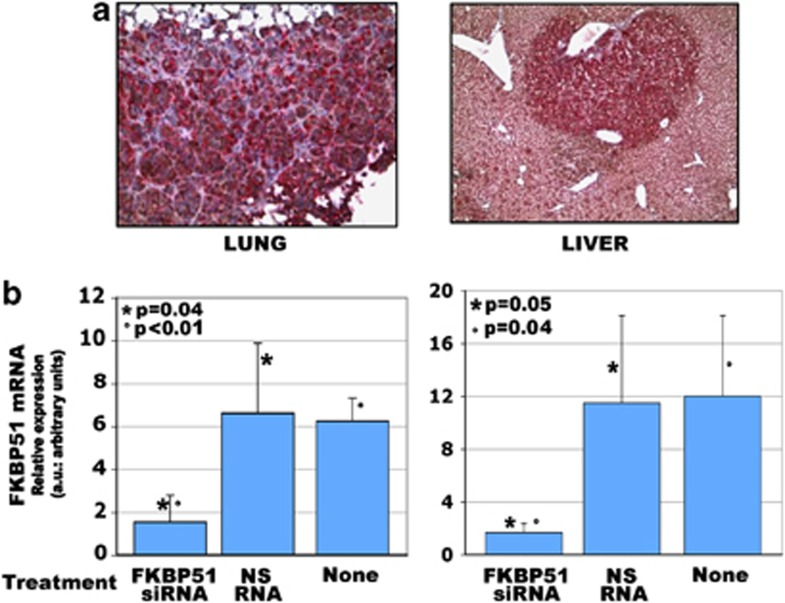
FKBP51 stains mouse metastasis. (**a**) FKBP51/nestin double-stain of melanoma cells in a lung and liver biopsy (IHC, × 200), from untreated mouse. (**b**) Expression levels of FKBP51 in mouse organs. RNA was extracted from a mix of multiple samples of tissue collected from the perihilar, apical, and basal areas of both lungs and from hilum and left and right lobes of livers. Graphic representation of normalized expression rates (mean and S.D.) of FKBP51 mRNA in lungs and livers excised from mice. A sample from FKBP51 siRNA-treated mouse was arbitrarily used as reference sample (expression=1). Expression of FKBP51 was significantly reduced in organs of mice receiving FKBP51 siRNA

Post-mortem organ examination showed a dramatic difference between the morphologically preserved organs dissected from FKBP51 siRNA-treated mice, and severely compromised organs from untreated or NS RNA-treated mice (Figure 4b, left). The histological examination confirmed that melanoma metastasis extensively substituted the lungs and liver of untreated or NS RNA-treated animals (Figure 4b, right). In contrast, few tumoural aggregates could be visualized in the lungs and livers of FKBP51 siRNA-treated mice (Figure 4b, right). Most interestingly, invading cells coexpressed FKBP51 and nestin (Figure 5a). A quantitative estimate of organ invasion was performed and is shown in Figure 5b. Measurement of FKBP51 transcript levels in lungs and livers confirmed reduced melanoma colonization of FKBP51 siRNA-treated mice.

### FKBP51 binds to chromatin

**Figure 6:**
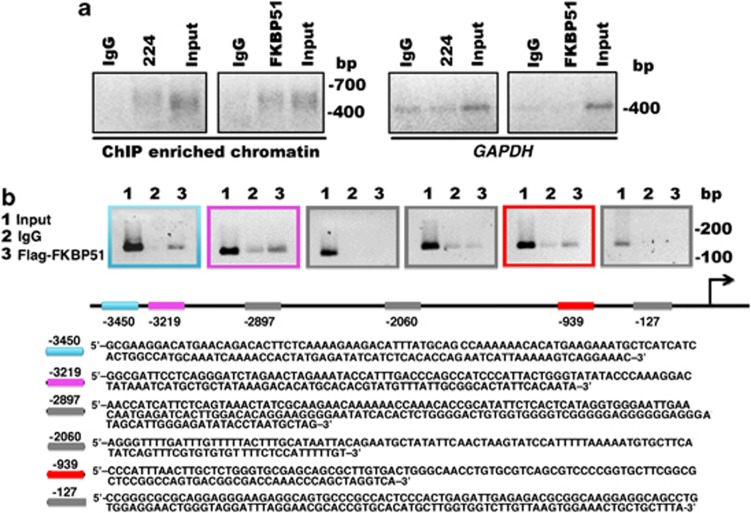
FKBP51 binds to *ABCG2* promoter. (**a**) Enriched DNA amplification bands in FKBP51-immunoprecipitated samples compared with IgG samples. ChIP with ZNF 224 served as positive control. ChIP was conducted with crosslinked chromatin, and DNA enrichment was evaluated using primers recognizing DNA-ligated poly-linkers (see Materials and Methods). G3PDH was used as ChIP quality control. (**b**) Enrichment of DNA regions at −3450 (blue square), −3219 (pink square), and −939 (red square) from TSS of *ABCG2* gene in FKBP51-immunoprecipitated chromatin. Specificity of FKBP51 binding to *ABCG2* promoter is suggested by lack of enrichment of DNA regions at −2897, −2060, and −127

**Figure 7:**
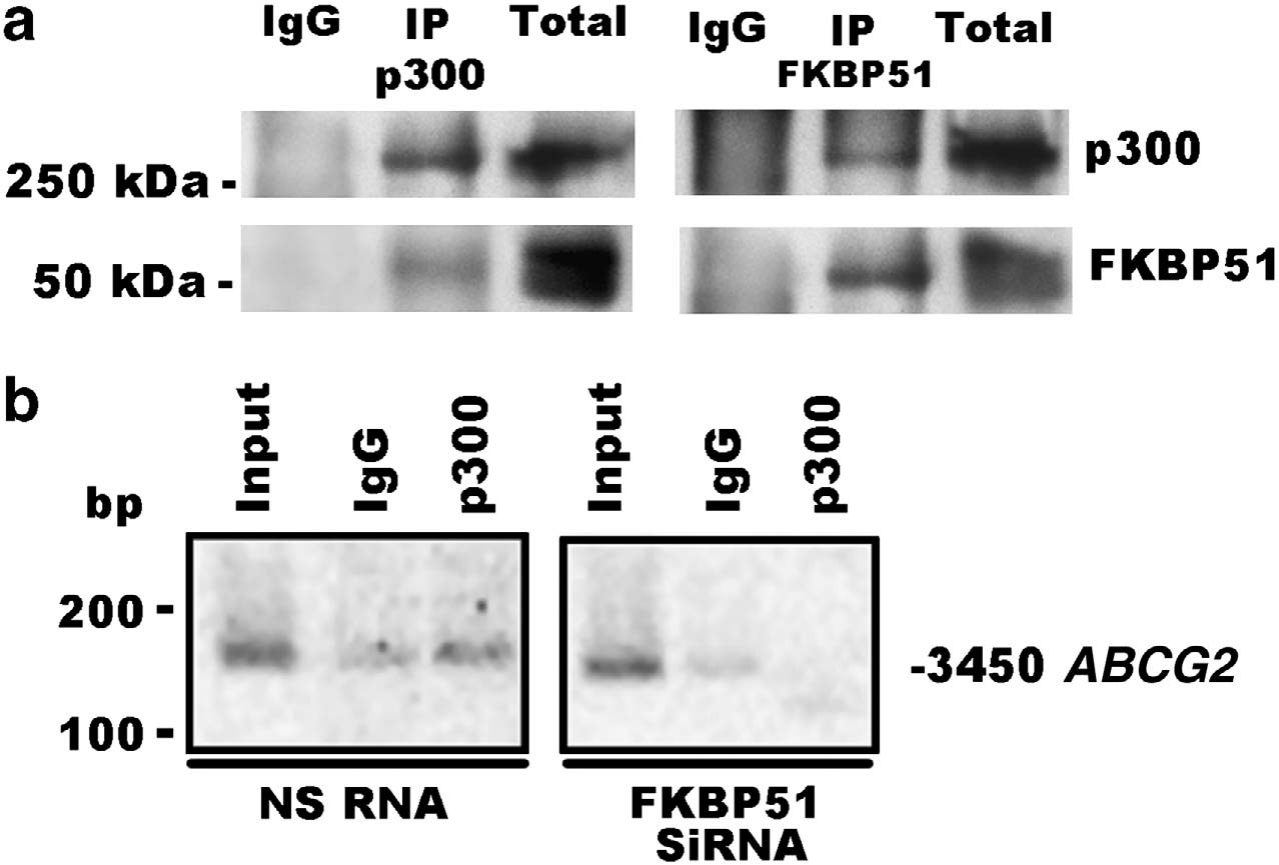
FKBP51 interacts with the general transcriptional co-activator p300. (**a**, left) FKBP51 co-immunoprecipitates with p300. (**a**, right), p300 co-immunoprecipitates with FKBP51. Total-cell lysates were prepared by SAN melanoma cells transfected with FKBP51/Flag. Cell lysates were immunoprecipitated with anti-Kat3B/p300 (IP p300) or anti-Flag (IP FKBP51). Immunoprecipitated and total lysates were then subjected to western blot with anti-FKBP51 or anti-p300. (**b**) ChIP performed with SAN melanoma cells, silenced (FKBP51 siRNA) or not (NS RNA) for FKBP51. An enrichment of DNA (region at −3450 from TSS of *ABCG2* gene) can be observed in p300-immunoprecipitated chromatin (NS RNA) compared with IgG sample. Such an enrichment appeared to be reduced when FKBP51 was silenced (FKBP51 siRNA)

Several lines of evidence support a role for FKBPs as transcriptional coregulators (reviewed in Yao *et al.*^15^). As we have found that FKBP51 modulated expression of several genes at transcriptional level, we hypothesized a role for FKBP51 as a transcriptional coregulator. To investigate whether the immunophilin interacted with DNA, we performed chromatin immunoprecipitation assays (ChIP) using anti-Flag antibody in melanoma cells transfected with FKBP51-Flag or the zinc-finger protein ZNF224-Flag^37^ as positive control. A representative result is shown in Figure 6a. We found enriched DNA amplification bands in anti-Flag-immunoprecipitated samples, which suggested that FKBP51, similar to the zinc-finger protein, binds to chromatin. We then attempted to address whether the FKBP51-IP chromatin included a promoter of a gene, whose expression appeared to be regulated by the immunophilin. Among the regulated genes, we choose *ABCG2*, because the transcript of this gene was found very abundant in melanoma metastasis samples from patients. We constructed primers to amplify different regions of ABCG2 promoters. Our results showed enrichment in DNA fragments, size selected on an agarose gel, corresponding to three different regions of *ABCG2* promoter: the first localized at 3450 bp (−3450) from the transcription start site (TSS), the second at −3219, and the third at −939 in FKBP51-immunoprecipitated chromatin (Figure 6b). Differently, no enrichment of −2897, −2060, and −127 regions was found, which is consistent with the specificity of FKBP51 binding (Figure 6b). This result suggests an interaction between FKBP51 and *ABCG2* promoter. Because results from stem cell array (shown in Supplementary Figure S5, gene 8) have suggested that ABCG2+ cells contained increased levels of the general transcriptional co-activator, the E1A binding protein 300 (p300), we investigated whether FKBP51 interacted with this protein, which reinforced our hypothesis that immunophilin takes part in transcriptional complexes. A representative result of three independent experiments is shown in Figure 7a. We found that FKBP51 co-immunoprecipitated with p300, and, conversely, p300 co-immunoprecipitated with FKBP51, providing evidence that the two proteins interact with each other. In addition, we found DNA enrichment corresponding to the −3450 sequence of *ABCG2* promoter in p300-immunoprecipitated chromatin, which suggested that FKBP51 and p300 participated in the same transcriptional complex (Figure 7b). Interestingly, such enrichment was not observed in FKBP51-silenced cells, which suggests FKBP51 favours interaction between p300 and DNA. Overall, these results are consistent with our hypothesis that FKBP51 may act as a transcriptional coregulator.

## Discussion

Melanoma often starts as a single skin tumour. Following primary surgical resection, this tumour can remain clinically quiescent for years but, unpredictably, malignant melanocytes can spread to distant locations and cause metastasis. The most common sites of melanoma metastases are in the subcutaneous tissue, lymph nodes, lungs, liver, brain, and bone. In most cases, there is no cure for advanced melanoma. The change during melanoma progression, that causes the switch from a locally growing tumour to a metastatic invader, remains obscure. In the present study, we show that FKBP51 plays a major role in promoting activation of genes involved in melanoma progression.

FKBP51 increased expression of TGF-*β* and, consistent with the notion that this cytokine is the master regulator of EMT,^24, 25, 26^ immunophilin promoted EMT features and improved migration and invasiveness of melanoma cells. In addition, FKBP51 regulated expression of MCSC markers at transcriptional level and the amount of malignant melanocytes with a cancer stem cell phenotype resulted significantly increased when FKBP51 was overexpressed. Most interestingly, melanoma cells with cancer stem cell phenotype expressed higher levels of EMT gene transcripts in comparison with the rest of the melanoma cells. Conversely, metastatic melanoma expressed CSC markers at higher levels in comparison with primary melanoma. These findings suggest a strict relationship between MCSC phenotype and melanoma invasiveness, in accordance with a previous study.^29^ Notably, appearance of stemness markers on melanoma cells can occur within reversible changes in a broad range of markers, including EMT markers, that turn on and off within tumour in a context of phenotypic heterogeneity and plasticity.^3, 23^ Several mechanisms, for example, drug resistance,^5, 6, 8, 38^ immune evasion,^39^ and vasculogenic mimicry,^40^ confer aggressiveness to these cells. In this perspective, FKBP51 could be an important regulator of melanoma plasticity. The finding that FKBP51 participates in transcriptional complexes supports this conclusion. Moreover, the interaction with p300 acetyltransferase is also in accordance with a role for FKBP51 in the chromatin changes required for melanoma cell reprogramming. The translational significance of our findings was strengthened by immunochemical evaluation of primary and metastatic melanoma specimens, suggesting that coexpression of FKBP51 and the stem cell marker nestin is a distinctive feature of the invading melanoma cell, which is equipped for extravasation, easily adaptable to the colonized milieu, and capable of subverting the microenvironment of distant sites. In fact, in primary melanoma, malignant melanocytes coexpressing FKBP51 and nestin become clearly visible in the intradermal, invasive growth phase; the same double-stained cells formed neoplastic emboli in dermal vessels of skin lesions. In advanced melanoma, malignant melanocytes in vascular and perivascular compartments were FKBP51/nestin double positive. Finally, malignant melanocytes totally subverting the brain tissue showed the same immunochemical feature. In an experimental model of melanoma metastasis, FKBP51 and nestin were coexpressed in malignant melanocytes that colonized murine organs. The important role of FKBP51 in melanoma progression was reinforced by studies in this mouse model of metastatic melanoma using FKBP51siRNA as a therapeutic approach. *In vivo* imaging and anatomo-histopathological examination inequivocally showed that FKBP51 targeting dramatically reduced metastases in mice.

In conclusion, our study suggests that FKBP51 may be a suitable novel melanoma biomarker that can improve the risk classification of primary lesions. In addition, we provide a promising target for development of compounds that can prevent metastasis after surgical treatment, forecasting that prognosis of this unpredictable, insidious tumour, which is lethal in advanced stages, may change in the very near future.

## Materials and Methods

### Cell culture and transfection

The melanoma cell lines SAN, A375, G361, and SK-MEL-3 were cultured as described.^8^ To create FKBP51-overexpressing SAN melanoma cells, a p3XFLAG-CMV-14 expression vector (Sigma Aldrich, St. Louis, MO, USA) carrying the FKBP51 gene was transfected using Metafectene (Biontex, Munich, Germany), according to the manufacturer's recommendations. A void p3xFlag-CMV vector was also transfected to generate control cells. To generate stable populations, cells were selected using 500 *μ*g/ml G418 (GIBCO, Invitrogen, Carlsbad, CA, USA) at 24 h post transfection and grown until colony formation. Different clones were expanded and analysed by western blot using M2 anti-FLAG antibody (Sigma Aldrich) and real-time PCR using specific primers for FKBP51 (see real-time PCR section for primer sequences); three of these clones were chosen for further manipulation. For siRNA transfection, 24 h before transfection, cells were seeded into six-well plates at a concentration of 2 × 105 cells/ml to obtain 30–60% confluence at the time of transfection. Then, cells were transfected with specific short-interfering oligoribonucleotide (siRNA) or with a NS oligoribonucleotide (NS RNA) as control at a final concentration of 50 nM using Metafectene according to the manufacturers' recommendations. NS RNA (AllStars neg control siRNA) and siRNA for FKBP51 (5′-ACCUAAUGCUGAGCUUAUA-3′) were purchased from Qiagen. In experiments shown in Supplementary Figure S2 and S3, the sequence 5′-GCCGAUGAUUGGAGACAAA-3′ (Dharmacon Thermo Scientific, Chicago, IL, USA) of FKBP51 siRNA (siRNA 2) was used.

### FKBP51 cloning

A DNA fragment encoding the complete FKBP51 protein was amplified from PBMC cDNA, with the 5′ forward: GGAATTCCGGCTGAAGGGTTAGCGGAGCAC and the 3′ reverse: CGGGATCCCGTACGTGGCCCTCAGGTTTCTCTTC. The amplified fragments were cloned into *Eco*RI/*Bam*HI sites of p3XFLAG-CMV-14. Half of the ligation was transformed into *E.Coli Top-ten* high-competence bacteria. After 16–20 h of growth at 37°C, 10–25 colonies/dish were obtained and expanded in L-Broth (5 ml) containing 50 *μ*g/ml ampicillin. Clones containing inserts were subjected to digestion with *Eco*RI and *Bam*HI, and to triple digestion with *Eco*RI, *Hin*dIII, and *Bam*HI, to obtain FKBP51 cleavage in three differently sized fragments. Clones were sequence-verified by Primm (Milano, Italy).

### Western blot and immunoprecipitation

Whole-cell lysates were homogenized in modified RIPA buffer as described^9^ and assayed in western blot as described.^9^ Primary antibodies against FKBP51 (F-13; goat polyclonal; Santa Cruz Biotechnology, Santa Cruz, CA, USA), TGF-*β* (V; rabbit polyclonal; Santa Cruz Biotechnology), and Smad 2/3 (H465, rabbit polyclonal Santa Cruz Biotechnology) were used diluted 1 : 500; anti-FKBP51 (rabbit polyclonal; Novus Biologicals, Littleton, CO, USA), anti-FKBP51 (mouse polyclonal; Abnova, Taipei, Taiwan), anti-FKBP12 (N-19, goat polyclonal; Santa Cruz Biotechnology), anti-actin (I-19, goat polyclonal; Santa Cruz Biotechnology), anti-G3PDH (D16H11; rabbit monoclonal; Cell Signaling, Danvers, MA, USA), and anti-KAT3B/p300 (RW109; mouse monoclonal, Novus Biologicals) were used 1 : 1000. Anti-ABCB5 (rabbit polyclonal; Rockland, Gilbertsville, PA, USA) was used 1 : 5000. For immunoprecipitation (IP), 500 *μ*g of total lysate was precleared for 1 h. Then, 3 *μ*g anti-KAT3B/p300 (Novus Biologicals) or anti-Flag (M2, mouse monoclonal, Sigma Aldrich) was added to total lysate, and kept in rotation at 4°C overnight. Next, 25 *μ*l protein A/G plus-Agarose (Santa Cruz Biotechnology) was added to the mixture and precipitation took place for 4 h, with rotation at 4°C. Samples were then washed in RIPA and separated by 10% SDS-PAGE.

### Real-time PCR

Total RNA was isolated from cells using Trizol (Invitrogen, Carlsbad, CA, USA) and from paraffinized tissues using the High Pure RNA Paraffin Kit (Roche Diagnostics GmbH, Mannheim, Germany) according to the manufacturer's instructions. One microgram of each RNA was used for cDNA synthesis with Moloney Murine Leukemia Virus Reverse Transcriptase (M-MLV RT, Invitrogen). Gene expression was quantified by Real-time PCR using iQSYBR Green Supermix (Bio-Rad, Hercules, CA, USA) and specific real-time validated QuantiTect primers for *FKBP5* (QT00056714: NM_001145775 800 e 900 bp; NM_001145776 650 e 750 bp; NM_001145777 650 e 750 bp; NM_004117 600 e 700 bp), *ABCG2* (QT00073206: NM_0048271100 e 1200 bp), *TGFB1* (QT00000728: NM_0006601200 e 1300 bp), *ENG* (QT00013335: NM_000118600 e 750 bp; NM_001114753600 e 750 bp), *VIM* (QT00095795: NM_003380 900 e 1000 bp), *SNAI2* (QT00044128: NM_003068200 e 350 bp), *SNAI1* (QT00010010: NM_005985600 e 750 bp), *TWIST1* (QT00011956: NM_00474900 e 1000 bp), *CDH1* (QT00080143: NM_004360700 e 800 bp), *CDH2* (QT00063196: NM_0017922800 e 2900 bp) (Qiagen), and ribosomal 18S primers Fw 5′-CGATGCGGCGGCGTTATTC-3′ and 18S Rev 5′-TCTGTCAATCCTGTCCGTGTCC-3′. Relative quantitation of the transcript was performed using co-amplified ribosomal 18S as an internal control for normalization.

### Cell proliferation, soft agar colony formation, and invasion assays

Cell proliferation was measured using a Cell Counting Chamber and Trypan blue. Briefly, cells were seeded onto a 24-microwell plate (15 × 103/well). After a 2-day culture, cells were added with Trypan blue (0.8 mM in PBS) to stain dead cells, and loaded onto a Burker's counting chamber, covered with a cover slide, and then counted under a microscope. The soft agar colony formation assay was done in 6 cm plates. A bottom layer of a solution containing Dulbecco's modified Eagle's medium 2 × (Sigma, St. Louis, MO, USA), TPB Buffer (Difco, BD, Franklin Lakes, NJ, USA), and 1.25% of Noble Agar (Difco) was poured first (7 ml); after solidifying, this was followed by a layer containing 2 ml of the same solution and 5 × 104 cells. The plates were placed in the incubator and after 10 days, colonies were photographed and counted. The invasion assay was performed in accordance with Albini and Benelli.^28^ Briefly, this method is a simple modification of Boyden chamber assay because of addition of matrigel, which mimics the physiological barrier to tumoural cells, allowing to evaluate both migratory and invasive capabilities of tumoural cells. Transwell chambers, with 8 *μ*m polyester filters, were used for this assay (Corning Incorporated Life Sciences, Tewksbury, MA, USA) and coated with 100 *μ*l of basement membrane matrix, matrigel (Becton Dickinson, Franklin Lakes, NJ, USA). The matrigel was diluted with cold PBS 1 × , applied to the filters, dried in humidified atmosphere at 37°C for at least 5 h, and reconstituted with serum-free medium. The coated filters were placed in Boyden chambers, forming two medium-filled compartments separated by such microporous membrane. A nutrient gradient was created between the two chambers (1% FCS medium in the upper and 10% FCS medium in the lower compartment). Melanoma cells (75 × 103) were placed in the upper compartment and, after a 48-h incubation time, the membrane between the two compartments was fixed and stained with 0.25% Crystal Violet in methanol. Cells at the lower surface of the filter were photographed; then, the Crystal Violet was eluted in 1% SDS and read in a spectrophotometer at a wavelength of 570 nm.

### Flow cytometry

Endoglin and ABCG2 expression were measured using the mouse monoclonal antibodies anti-CD105-phycoerythrin (PE) (eBioscience, San Diego, CA, USA) and anti-ABCG2-PE (R&D Systems, Minneapolis, MN, USA) and flow cytometry, as described previously.^8^ ABCG2+ cells were sorted from melanoma cell lines with a BD FACSAria (BD Biosciences, San Jose, CA USA). The population sorted was >80% ABCG2+.

### Immunohistochemistry

The procedures followed for immunohistochemistry studies on human samples were in accordance with the ethical standards of the local institutional responsible committee on human experimentation and with the Helsinki Declaration of 1975, as revised in 1983. Immunostaining for FKBP51 and nestin was performed using a dual chromogen method (peroxidase with brown chromogen followed by alkaline phosphatase (AP) with red chromogen). Serial sections from paraffin-embedded tumours were cut and deparaffinized as previously described.^8^ To inhibit nonspecific antibody binding, slides were treated with 3% normal goat serum before incubation for 1 h at room temperature with anti-FKBP51 primary antibody (sc-13983, Santa Cruz Biotechnology) diluted 1 : 200, anti-nestin (sc-23927, Santa Cruz Biotechnology) diluted 1 : 200, or anti-MART-1 (melanoma antigen recognized by T cells-Melan A. A103, Ventana Medical Systems Inc., Tucson, AZ, USA). Signals were amplified using the appropriate biotinylated secondary antibody, and detected with diaminobenzidine/H2O2 for peroxidase or Fast Red with naphthol phosphate substrate for AP (EnVision G/2 Doublestain System, Rabbit/Mouse (DAB+/Permanent Red), Dako, Glostrup, Denmark). For each run, sections from normal (reactive) human lymph node and glioma were respectively used as positive control for FKBP51 and nestin. For negative controls, nonimmune serum (1 : 500) in Tris-buffered saline (TBS) buffer was used instead of the primary antibodies. The immunohistochemical evaluation was performed independently by two consultant pathologists (SS and MM). The cells were judged positive for FKBP51 when a brown signal confined to the nucleus and for nestin when a red cytoplasmatic/membrane staining was observed. In all cases, 10 fields ( × 40 magnification) were assessed, evaluating a minimum of 100 cells in the selected (representative) areas. Sections were counterstained with Mayer's haematoxylin.

### ChIP assay

ChIP was conducted with crosslinked chromatin, prepared as described previously.^37^ Briefly, human SAN melanoma cells were transfected with a vector carrying the zinc-finger protein ZNF224 cloned into p3XFLAG-CMV (as positive control for chromatin binding) or with 3XFLAG-FKBP51. The fixed DNA was sheared by sonication, diluted, and precleared with protein A/G plus-agarose (Santa Cruz Biotechnology). The precleared extract was divided into two aliquots: one was used for control IgG and the other was immunoprecipitated with anti-Flag (3 *μ*g). IP-DNA was purified by A/G plus-agarose protein binding and crosslinks were reversed at 65°C overnight. DNA was then purified by phenol–chloroform extraction. After purification, 5 ng of immunoprecipitated DNA was subjected to ligation with linkers, as described previously.^26^ Briefly, the linkers consisted of a 24-mer oligonucleotide of sequence 5′-AGAAGCTTGAATTCGAGCAGTCAG-3′ annealed with a 20-mer of sequence 5′-CTGCTCGAATTCAAGCTTCT-3′, of which only the 24-mer is phosphorylated at the 5′ end. After ligation, amplification was carried out directly in a 50-l reaction using Taq polymerase (EconoTaq, Lucigen, Middleton, WI, USA) and the corresponding buffer, deoxynucleotides at 250 *μ*M, and 1 *μ*M primer (the 20-mer oligonucleotide specified above). The amplification cycles were 1 cycle of 94°C for 2 min; 35 cycles of 94°C for 1 min, 55°C for 1 min, 72°C for 2 min; and 1 cycle of 72°C for 5 min. For amplification of ABCG2 promoter fragments, the following couples of primers were utilized:

**−***3450*FW 5′-ACAACCCCATCAAAAAGTGG-3′

REV 5′-CATCCTCTCCAGCACCTGTT-3′

*−3219*FW 5′-ACCATTGTGGAAGTCGGTGT-3′

REV 5′-GGTTGGTTCCAAGTCTTTGC-3′

**−***2897*FW 5′-GGGACATGGATGAAATTGGA -3′

REV 5′-TGCACCCACTAACGTGTCAT-3′

**−***2060*FW 5′-CGTGCCTGGCCTCTATGTAT-3′

REV 5′-GGCACTACAGGAGGAGACTGA-3′

*−939*FW 5′-ATCCCATTCACCAGAAACCA-3′

REV 5′-GGGCTGATCAGTACCTCGTC-3′

*−127*FW 5′-CTGTGCCCACTCAAAAGGTT-3′

REV 5′-CGGACCTTCCAAACAAACTC-3′

The amplification cycles were 1 cycle of 94°C for 5 min; 35 cycles of 94°C for 1 min, 58°C for 30 s, 72°C for 2 min; and 1 cycle of 72°C for 5 min. The p300- immunoprecipitated chromatin was obtained using KAT3B/p300 Antibody (RW109) (Novus Biologicals).

### Animal studies

After the approval of the local institutional animal research committee, animal studies were performed following detailed internal regulations devised according to the US Public Health Service Policy on Humane Care and Use of Laboratory Animals, available from the Office of Laboratory Animal Welfare, National Institutes of Health, Department of Health and Human Services (Bethesda, MD, USA) and the United Kingdom Coordinating Committee on Cancer Prevention Research's Guidelines for the Welfare of Animals in Experimental Neoplasia (published online 25 May 2010). Melanoma cells (1.5 × 106 SAN in 100 *μ*l PBS) were injected systemically into the lateral tail vein of 4- to 6-week-old Balb/c IL2 NOD SCID (null) mice (Charles River Laboratory, Wilmington, MA, USA). Eight mice received treatment 15 and 21 days after SAN cell injections and four mice received no treatment. Treatment consisted of FKBP51 siRNA (four mice) or NS RNA (four mice) as control (100 *μ*g/mouse), systemically injected into the tail vein. RNA was delivered into liposomes (Metafectene), and resuspended in 100 *μ*l PBS.

### Imaging

Imaging was performed using a dedicated animal PET/CT scanner (eXplore Vista, GE Healthcare, Fairfield, CT, USA). A dose of 8.3 mCi/kg (307.1 MBq/kg) of [18]F-FDG was administered in a bolus in a total volume of 100 *μ*l. Animals were maintained at a temperature of 23°C during the biodistribution of [18]F-FDG. After 45 min, mice were anaesthetized with ketamine 50 mg/kg and xylazine 40 mg/kg and symmetrically positioned on a warm bed with micropore tape. Then, a 20-min static PET (two bed position with a 4.8-cm axial field-of-view; energy window 250–700 keV) scan was performed, followed by a 7-min CT scan. PET images were processed using a 2D-OSEM iterative algorithm (voxel size of 0.3875 × 0.3875 × 0.7750 mm3) including random scatter correction, dead time, decay, and attenuation correction using CT data (eXplore Vista Software). SUVs were calculated from the PET studies (SUV=tissue activity (MBq/kg)/(injected dose (MBq)/body weight).

### Statistical analysis

The statistical significance of differences between means was estimated using Student's *t*-test. Values of *P*<0.05 were considered statistically significant.

## Abbreviations

FKBP51: FK506 binding protein 51
MCSC: melanoma cancer stem cell
CSC: cancer stem cell
PAS-1: PASTICCINO1 gene
EMT: epithelial to mesenchymal transition
TGF-*β*: transforming growth factor *β*
T*β*RIII: type III TGF-*β* receptor
siRNA: small interfering RNA
ABCB5: ATP binding cassette transporters, subfamily B, member 5
ABCG2: ATP binding cassette transporters, subfamily G, member 2
EV: empty vector
MART-1: melanoma antigen recognized by T cells
CT: computerized tomography
PET: positron emission tomography
[18]F-FDG: Fluorodeoxyglucose (18F)
SUV: standardized uptake values
TSS: transcription start site
