# Supplementary material for: FK506 binding protein 51 positively regulates melanoma stemness and metastatic potential
Source: Cell Death Dis. 2013 Apr 4;4(4):e578–. doi: 10.1038/cddis.2013.109 (PMC3641332; doi:10.1038/cddis.2013.109)
Supplement: Supplementary Information [file cddis2013109x1.doc]

#### Legends to Supplementary Figures

**Fig S1** *Effect of* *FKBP51 silencing on migration and invasion of melanoma cells.* **A,** Western blot assay of cell lysates obtained from SAN melanoma cell line stably transfected with a specific FKBP51 shRNA, or a non-silencing (NS) shRNA as control. ShRNA transfection was performed by using the Expression Arrest^TM^ shRNA system (Open Biosystem, AL, USA). Expression Arrest^TM^ shRNAs are cloned into the incompetent replication pSHAG-MAGIC2 (pSM2) retroviral vector. This vector has a Murine Stem Cell Virus (MSCV) backbone combined with packaging extract for mammalian cell infection, a PGK-Puro selection for transfection stability in mammalian cells and a Chloramphenicol/Kanamycin Bacterial selection marker. The stable transfectants were obtained after a 1 month selection of positive clones, with puromycin (Sigma Aldrich, Saint Louis, Missouri, USA). For a first stronger selection puromycin was used at a dose of 800 ng/ml; after a week it was used at 350ng/ml. **B**, Boyden chamber filters stained with Crystal Violet. O.D. value was obtained by reading the eluted Crystal Violet with a spectrophotometer at a wavelength of 570 nm. Upper, transwell migration of non silenced (NS-Sh) and silenced (FKBP51-Sh) melanoma cells. Lower, invasion of matrigel-coated filters by the same cells. The reduced O.D. in FKBP51-Sh samples indicated that FKBP51-silenced cells migrated and invaded matrigel less efficiently than control cells. The data represent the means of three independent experiments performed in triplicate.

**Fig S2** *Effect of* *FKBP51 silencing on EMT features, in A375 melanoma cell line*. For FKBP51 silencing of A375, the siRNA2 (see Materials and Methods) was used. **A**, Normalized expression rates (mean+s. d.) of FKBP51, TGF-β and TβRIII mRNA levels; FKBP51 siRNA-treated sample expression=1. **B**, Soft agar growth of A375 melanoma cells, silenced or not for FKBP51. Colony number and size of A375 was clearly reduced by FKBP51 silencing, in comparison to cells treated with a non silencing RNA. **C,** Boyden chamber filters invaded by A375 melanoma cells. Filter was stained with Crystal Violet. O.D. value was obtained by reading the eluted crystal violet with a spectrophotometer at a wavelength of 570 nm. FKBP51 silencing reduced filter invasion.

**Fig S3** *Effect of FKBP51 silencing on ABCB5 expression in different melanoma cell lines*. Western Blot assay of FKBP51 and ABCB5 expression levels in A375, SAN, SKMEL3 and G361 melanoma cell lines. For FKBP51 silencing, the FKBP51 siRNA2 (see Materials and Methods) was used.

**Fig S4** *Analysis of additional FKBP51 over expressing clones.* **A**, Real-time measurement of FKBP51 mRNA (black) and ABCG2 mRNA (grey) in WT, EV, and two clones over expressing FKBP51 (Ca, and Cf). For relative quantitation of transcript, WT sample expression=1. **B**, Western Blot assay of ABCB5 and TGF-β in WT, EV-, Ca and Cf. Anti-Flag marked exogenous FKBP51. **C**, Flow cytometric analysis of TβRIII (CD105) expression in Ca (left) and Cf (right). FKBP51-hyperexpressing cells (black line) moved to the right relative to WT or EV cells (grey line), indicating increased CD105 expression. Mean fluorescence intensity (MFI) of the samples is also shown.

**Fig S5** *Enhanced STEM CELL gene transcripts in sorted ABCG2^+^ melanoma cells.*

Total RNA was extracted from ABCG2^+^ and ABCG2^-^ cells, separated from SAN melanoma that over expressed FKBP51; RNA aliquots (2 ug for each sample) were reverse transcribed using RT2 First Strand Kit (Qiagen Sciences, Maryland, USA), according to the manufacturer's protocol. Real-time PCR was performed using the RT^2^ profiler PCR Array-Human Stem Cell signaling pathway (Qiagen Sciences) with the RT^2^ SYBR Green qPCR Mastermix (Qiagen Sciences) and carried out with the iCycler iQ Real-Time detection system (Bio-Rad Laboratories). Gene expression level was normalized to G3PDH and RPL13A as endogenous controls. Relative expression was calculated using ABCG2^-^ cells as reference sample (expression=1).

**Fig S6** *Efficacy and specificity of FKBP51 siRNA.* **A,** Efficacy of FKBP51 silencing: total lysates were prepared by SAN melanoma cells transfected with NS RNA or FKBP51 siRNA at the indicated times and analyzed by Western blot. FKBP51 downmodulation becomes detectable as soon as after 6h from transfection and persists at 72h from transfection. **B,** Specificity of FKBP51 siRNA: Western blot assay of total lysates prepared by SAN melanoma cells not transfected or transfected with NS RNA or FKBP51 siRNA. FKBP51 siRNA downmodulates FKBP51 but not FKBP12. **C,** In vivo efficacy of FKBP51 siRNA: Western blot assay of FKBP51 levels in lysates prepared from melanoma xenografts obtained from athymic nu/nu mice. When tumors reached 10 mm in mean diameter, mice received a single intratumoral injection of FKBP51 siRNA or NS RNA. After 96 h from injection, animals were sacrificed and tumors excised for preparation of lysates.
