# Supplementary figures and images for: FK506 binding protein 51 positively regulates melanoma stemness and metastatic potential
Source: Cell Death Dis. 2013 Apr 4;4(4):e578–. doi: 10.1038/cddis.2013.109 (PMC3641332; doi:10.1038/cddis.2013.109)

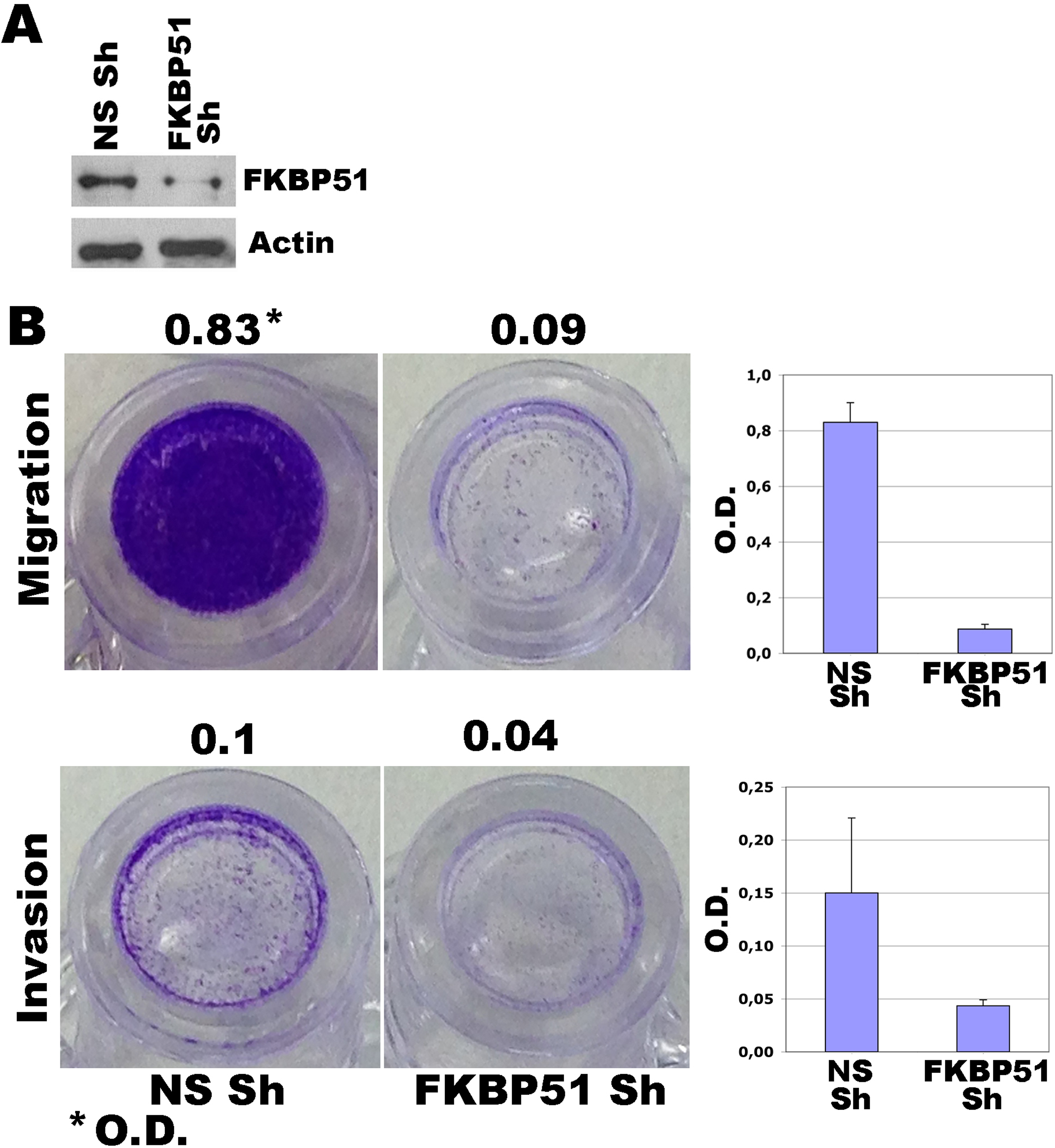

Supplement: Supplementary Figure S1 [file cddis2013109x2.tif]

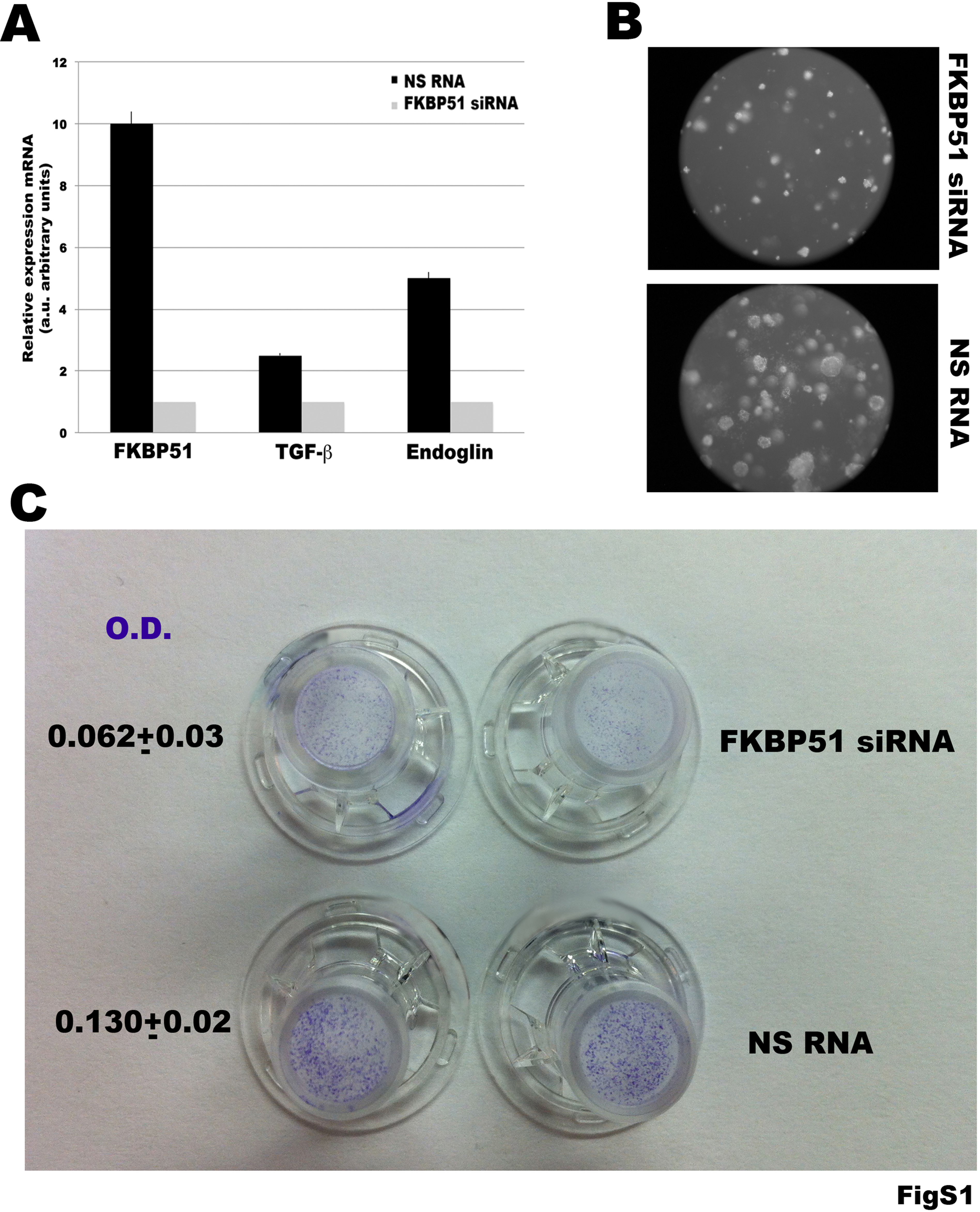

Supplement: Supplementary Figure S2 [file cddis2013109x3.tif]

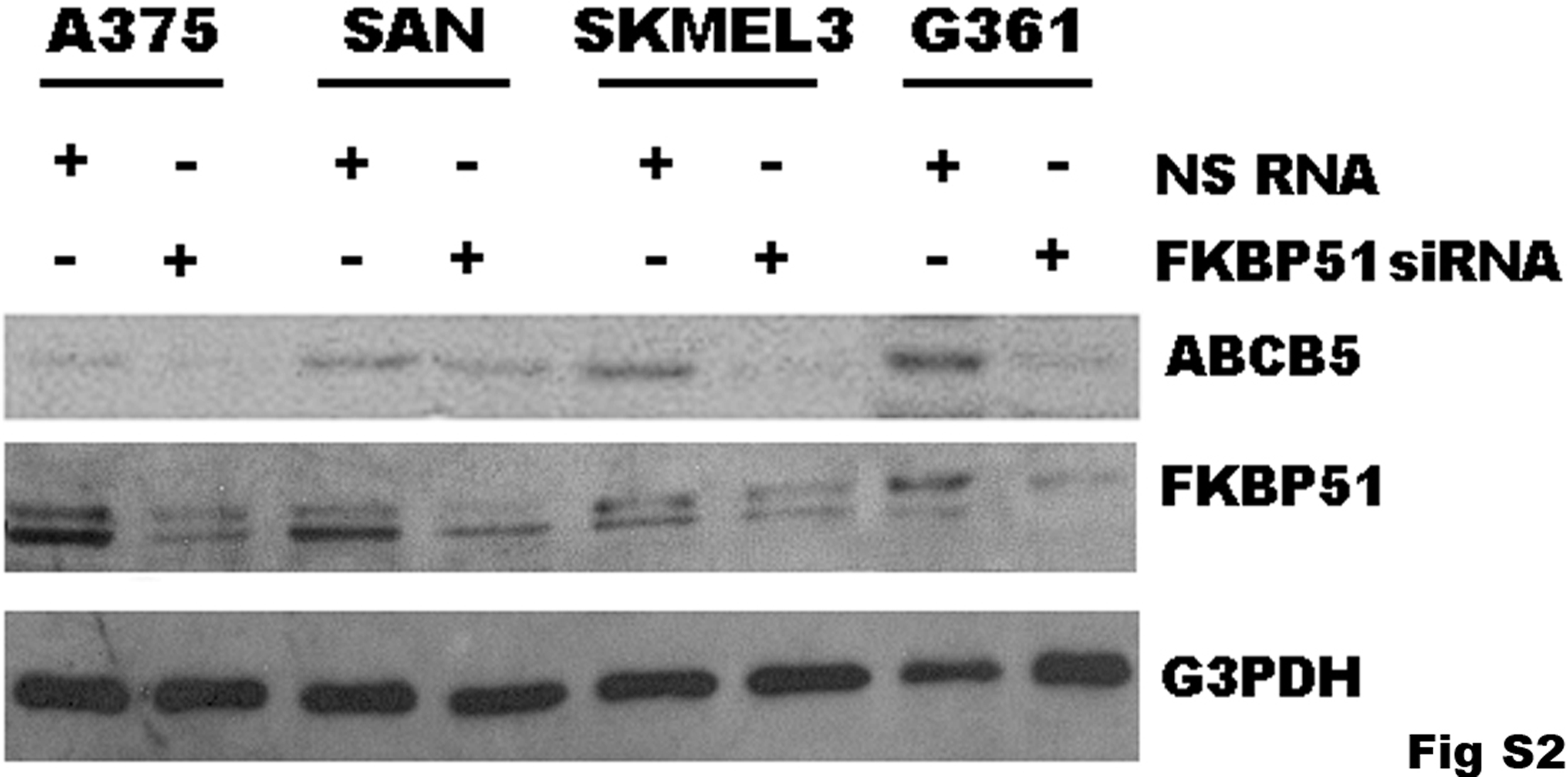

Supplement: Supplementary Figure S3 [file cddis2013109x4.tif]

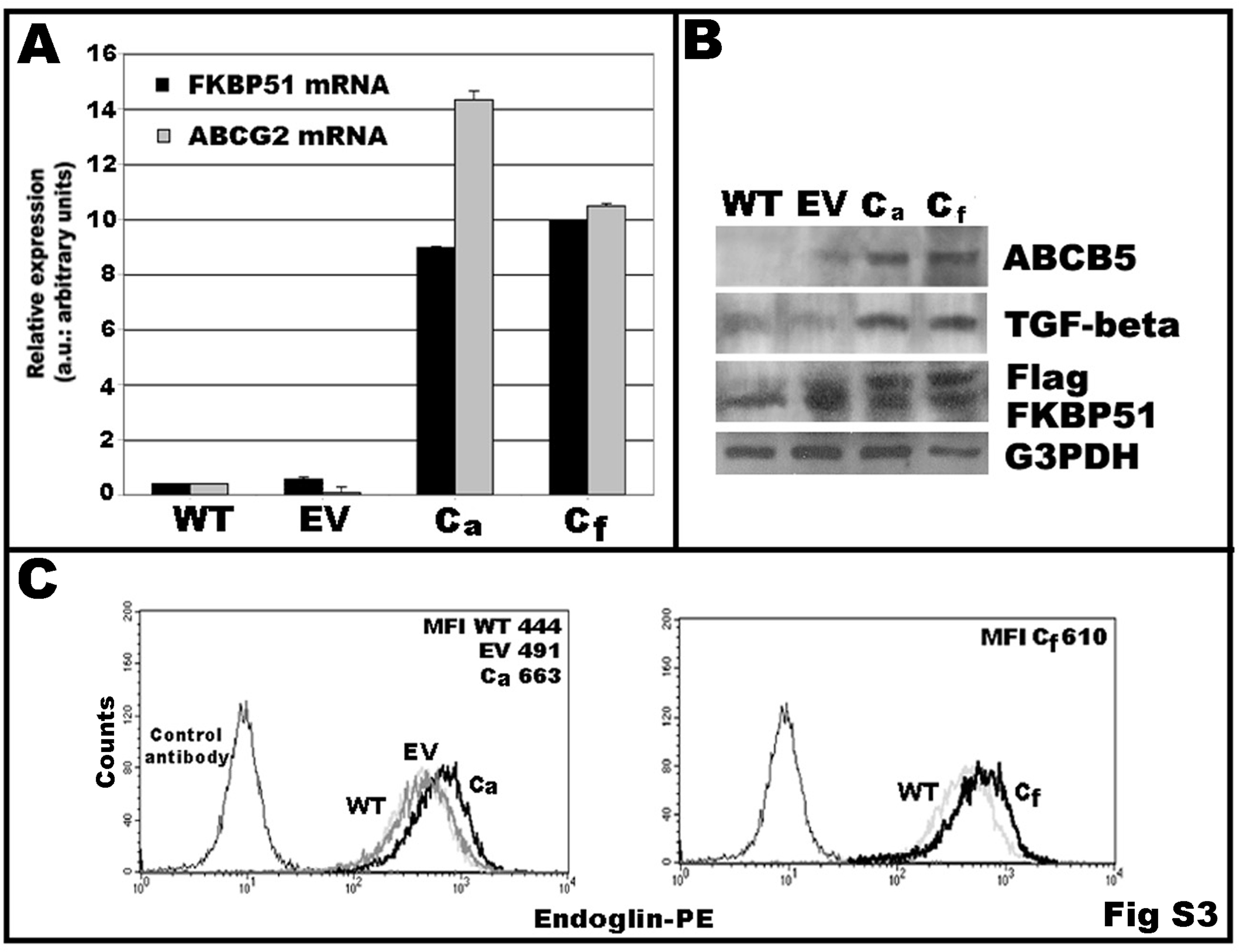

Supplement: Supplementary Figure S4 [file cddis2013109x5.tif]

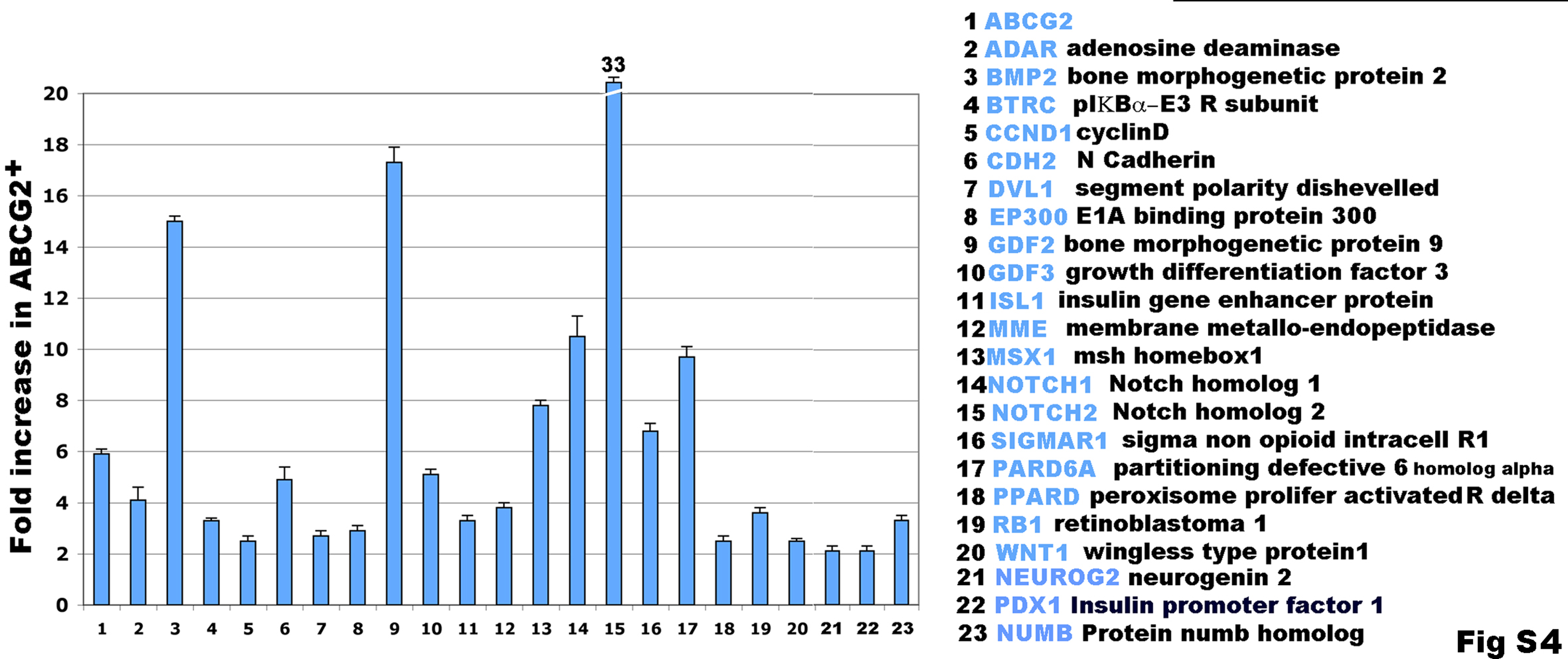

Supplement: Supplementary Figure S5 [file cddis2013109x6.tif]

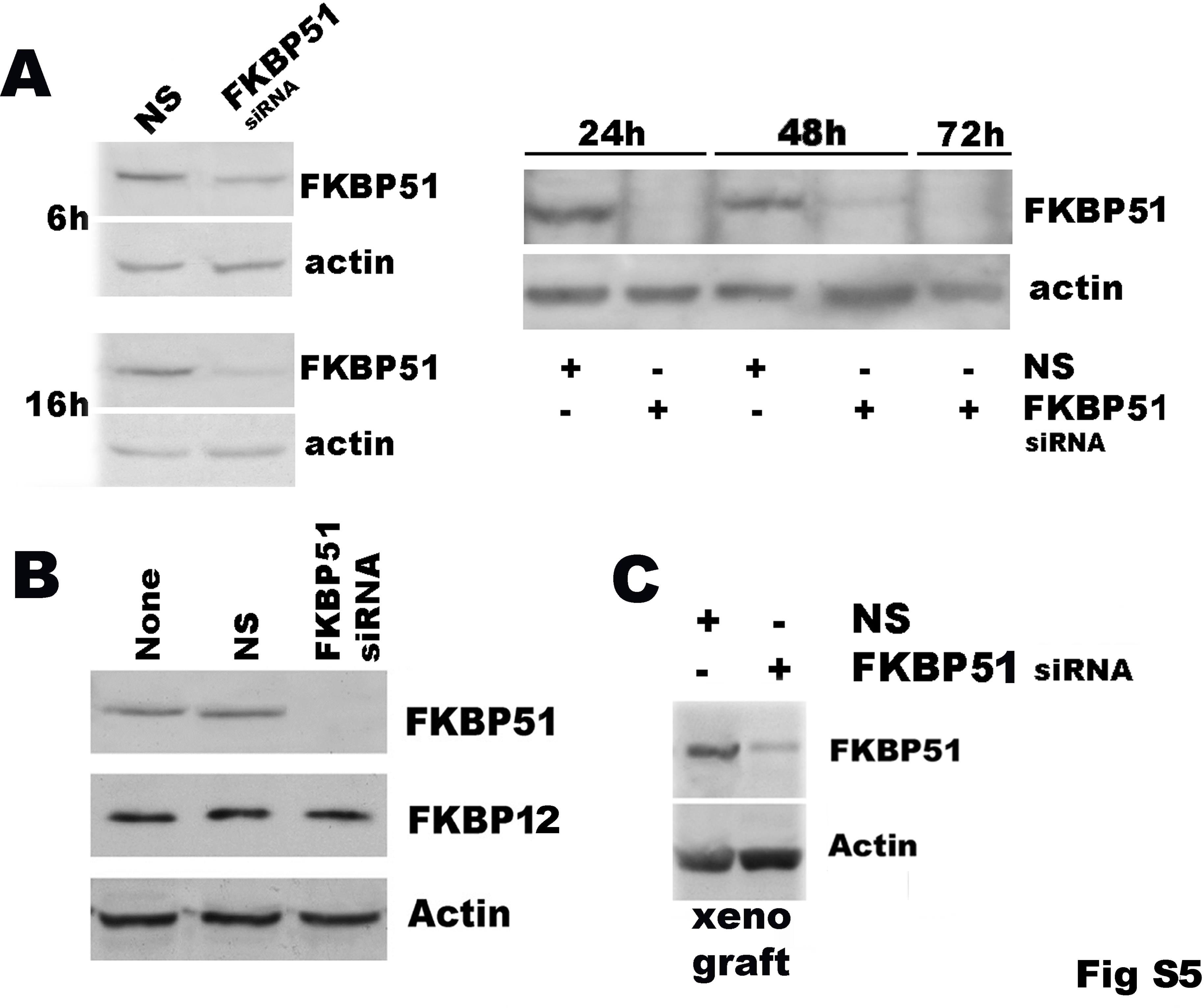

Supplement: Supplementary Figure S6 [file cddis2013109x7.tif]
